# Supplementary material for: Chromothripsis during telomere crisis is independent of NHEJ, and consistent with a replicative origin
Source: Genome Res. 2019 May;29(5):737–49. doi: 10.1101/gr.240705.118 (PMC6499312; doi:10.1101/gr.240705.118)
Supplement: Supplemental Material [file supp_gr.240705.118_Supplemental_file_1.zip › contigs/annotated_contigs/DB46/contig.2.DB46_length_470_mean_cov_8.87021276596.docx]

**DB46_length_470_mean_cov_8.87021276596**

```
AAGGGCAAGAGAATAGGAACATGTGCACACATGTGAGAATAGGAACATGTGAGGGAAAGAGAGACAGAACTAGATGCCTTAAGCCCTTT
 >chr14:86615102-86615338 + E=1e-122
TGTAATCAGCATTAATCCATCAAAGGTGGCTGAGCCCACATGACCTCAACACCTCTCAATAGACCCCACTTCTCAGCACTATTGCATTG

GGGATTATGTTTTATGTTTTCAACACATGCTTTAGGGAGGACACATTCAAACCATAGCAAG|GCCT|CTGAAACATACCCCTGTGTTCC
                                                              >chr14:86619159-86619379
TTATCTCTGAGGCTTTGTACTCTGCTCAGTTTCACGAGGTATAATTGTCTGTGATCTGTAGGATGCTAATTTAAATATCACCTACTTAT
+ E=8e-118
GGAGGTTTTATCCATTCACTCAGATGCTCTATTCCAGGACTTTTCTATTTAGAACTAACTCATTAGCTGATACTATTTAATCCATTTAA

TAGTGCCATCTTTCTT|CAAAATGAGGG
```
